# Exome sequencing pilot study in children with carbamazepine-induced serious skin reactions

**DOI:** 10.1186/2045-7022-4-S3-P119

**Published:** 2014-07-18

**Authors:** Ursula Amstutz, Casper Shyr, Neil H Shear, Michael J Rieder, Wyeth W Wasserman, Colin J Ross, Bruce C Carleton

**Affiliations:** 1 Inselspital University Hospital and University of Bern, Clinical Chemistry, Switzerland; 2 University of British Columbia, Medical Genetics,, and Centre for Molecular Medicine and Therapeutics, Canada; 3 University of Toronto and Sunnybrook Health Sciences Centre, Dermatology; Clinical Pharmacology and Toxicology, Canada; 4 University of Western Ontario, Schulich School of Medicine and Dentistry, Clinical Pharmacology, Canada; 5 University of British Columbia, Medical Genetics, and Centre for Molecular Medicine and Therapeutics, Canada; 6 University of British Columbia, Pediatrics and Medical Genetics, Canada; 7 University of British Columbia and British Columbia Children's Hospital, Pediatrics, Canada

## 

Stevens-Johnson syndrome (SJS) and drug-induced hypersensitivity syndrome (HSS, also known as DRESS) are life-threatening drug hypersensitivity reactions. Previous studies on genetic predictors for SJS and HSS were focused on immune-related genes and on known, frequent polymorphisms in the genome. For the anticonvulsant carbamazepine (CBZ), genetic markers for CBZ-induced SJS and HSS have been identified in the HLA region (HLA-B*15:02, HLA-A*31:01). However, many patients carriyng HLA-B*15:02 or HLA-A*31:01 tolerate CBZ, resulting in a low positive predictive value of a predictive test based on these genetic markers alone. In this pilot study, we aimed to identify rare genetic variants potentially associated with CBZ-induced SJS and HSS using whole exome sequencing in seven children with CBZ-induced SJS and five children with CBZ-induced HSS. Whole exome sequencing was performed using Illumina TruSeq exome capture and sequencing technology. Paired-end sequence reads were aligned and variants within 150 bp of the 64 Mb target region were identified using Bowtie, BWA and GATK. DNA samples from 95 CBZ-tolerant Canadian children were pooled and sequenced to a median depth of 300-fold to estimate frequencies of sequence variants identified in the hypersensitivity cases. All study participants were recruited through the Canadian Pharmacogenomics Network for Drug Safety. Children with CBZ-induced SJS or HSS were of European, Asian and First Nations ancestry with three SJS cases of Asian origin carrying HLA-B*15:02 and two HSS patients of European and First Nations origin carrying HLA-A*31:01. No individual rare single nucleotide variant (SNV) was strongly overrepresented in the SJS or HSS cases either in combined or in hypersensitivity reaction-specific analyses. Gene-based analyses revealed several candidate genes with rare nonsynonymous SNVs observed in a majority of SJS or HSS cases, respectively, which require follow-up in additional patients. With a hypothesis-free, comprehensive genetic screening approach, we identified new candidate genes and variants potentially involved in the development of CBZ-induced SJS or HSS for further investigation. The identified genes and pathways may lead to novel hypotheses for the pathomechanisms of these serious dermatologic reactions, potentially opening up new possibilities for prevention and treatment strategies.

